# Management of patients with acromegaly in clinical practice in the gulf countries: a Delphi consensus survey

**DOI:** 10.3389/fendo.2025.1593959

**Published:** 2025-06-12

**Authors:** Mussa H. Almalki, Tarik Elhadd, Khaled M. AlDahmani, Aishah Ekhzaimy, Abdullah Alqanaei, Hasan Frookh, Arwa Alyamani, Osamah Hakami, Zeinab Dabbous, Zaina Rohani, Wael Almistehi, Hazem Aljumah, Abdulla Alfutaisi, Alaaeldin Bashier, Moeber Mahzari

**Affiliations:** ^1^ Obesity, Endocrine, and Metabolism Center, King Fahad Medical City, Second Health Cluster Riyadh, Riyadh, Saudi Arabia; ^2^ College of Medicine, Alfaisal University, Riyadh, Saudi Arabia; ^3^ Endocrine Section, Department of Medicine, Hamad Medical Corporation, Doha, Qatar; ^4^ Division of Endocrinology, Department of Medicine, Tawam Hospital, SEHA, PureHealth, Al Ain, United Arab Emirates; ^5^ Adjunct Faculty, Department of Internal Medicine, College of Medicine and Health Sciences, United Arab Emirates University, Al Ain, United Arab Emirates; ^6^ Division of Endocrinology, Department of Medicine, Sheikh Tahnoon Bin Mohammed Medical City, SEHA, PureHealth, Al Ain, United Arab Emirates; ^7^ Endocrinology and Diabetes Unit, Department of Medicine, College of Medicine and King Saud University Medical City, King Saud University, Riyadh, Saudi Arabia; ^8^ Division of Endocrinology, Department of Medicine, Sabah Hospital, Kuwait, Kuwait; ^9^ Division of Endocrine & Diabetes, Salmanyia Medical Complex, Governmental Hospital, Ministry of Health, Manama, Bahrain; ^10^ Endocrine Division of Internal Medicine Department, King Abdullah Medical City in Holy Capital (KAMC-HC), Makkah, Saudi Arabia; ^11^ Division of Endocrinology, Department of Internal Medicine, Prince Mohammed bin Abdulaziz Hospital, Riyadh, Saudi Arabia; ^12^ Department of Medicine, College of Medicine, Sultan Qaboos University, Muscat, Oman; ^13^ Division of Endocrinology, Department of Medicine, Dubai Hospital, Dubai, United Arab Emirates; ^14^ College of Medicine, King Saud bin Abdulaziz University for Health Sciences, Riyadh, Saudi Arabia; ^15^ King Abdullah International Medical Research Center, Riyadh, Saudi Arabia; ^16^ Department of Medicine, Ministry of National Guard Health Affairs, Riyadh, Saudi Arabia

**Keywords:** gulf region consensus, delphi consensus, survey, acromegaly, treatment, surgery, radiotherapy

## Abstract

**Background:**

Acromegaly management practices in the Gulf region lack standardized guidelines, leading to variability in care. This study aimed to establish evidence-based regional consensus recommendations to address clinical challenges and align management with local healthcare resources.

**Methods:**

A three-round Delphi consensus survey was conducted among 15 endocrinology experts from six Gulf countries. Forty-six statements across six domains—primary treatment, pre-surgery treatment with somatostatin analogs (SSAs), second-line therapy, radiotherapy, post-surgery follow-up, and long-term management—were evaluated. Consensus was predefined as ≥66.8% agreement.

**Results:**

Strong consensus was achieved on surgical resection as first-line therapy for eligible patients (100% agreement), with referrals to multidisciplinary centers emphasized (93.8%). Preoperative SSAs were endorsed to reduce surgical/anesthesia risks in high-risk patients (93.8%). For second-line management, watchful waiting for asymptomatic patients with mildly elevated insulin-like growth factor-1 (IGF-1) (93.8%) and combination therapy (where feasible) were supported. Radiotherapy received unanimous agreement for specific cases. Structured post-surgical follow-up protocols, including biochemical testing timelines and remission criteria, were established. Long-term monitoring emphasized individualized risk assessment.

**Discussion:**

These guidelines provide a regionally tailored framework for acromegaly management, prioritizing surgery as the cornerstone of treatment while integrating adjuvant therapies and follow-up strategies aligned with Gulf healthcare infrastructures. The consensus reflects pragmatic adaptations to resource availability, such as endorsing watchful waiting in specific contexts. While acknowledging limitations such as potential expert bias, these consensus guidelines provide a framework for standardizing acromegaly care across the Gulf countries, with emphasis on surgical intervention as the cornerstone of treatment while recognizing the importance of adjunctive therapies.

## Introduction

Acromegaly is a rare but significant endocrine disorder typically caused by a growth hormone-secreting pituitary adenoma. The disease manifests with progressive physical changes such as enlarged hands, feet, and coarse facial features, and is often associated with comorbidities such as cardiovascular diseases, diabetes, hypertension, and joint disorders (1). Timely and effective diagnosis and management of acromegaly is crucial to prevent complications, and it typically involves a combination of surgery, pharmacotherapy (e.g., somatostatin analogs, growth hormone receptor antagonists, and dopamine agonists), and occasionally radiotherapy (2, 3).

Pituitary surgery, especially via the transsphenoidal approach, is a key method for achieving biochemical remission in treating pituitary adenomas. If surgery is unsuccessful or not an option, medical treatments are considered (4). First-generation somatostatin analogs (SSA) are widely used as first line medical option and provide adequate disease control in 30-50% of cases, with some patients experiencing adenoma shrinkage (5). Pegvisomant, a growth hormone (GH) receptor antagonist, is used when first-generation SSAs are not enough to control the disease (6). Pasireotide long-acting release (LAR), a second-generation SSA, can be effective in those who do not respond to first-generation SSAs but may cause significant hyperglycemia (7). Dopamine agonists are moderately effective and are used primarily in mild cases or as add on in a combination therapy (3). Focused radiotherapy is a last resort for patients who are not controlled after surgery and/or medical therapy (4).

Optimal treatment can be challenging in areas with limited healthcare resources, which is often the case in many regions, including the Arabian Gulf region.

In the Gulf region, the treatment of acromegaly varies considerably due to differences in healthcare infrastructure, availability of specialized care, health insurance coverage and access to new medical therapies (8, 9). Moreover, the availability of advanced surgical interventions and radiotherapy can vary, with some countries facing limitations in access to these services, which may delay optimal treatment and outcomes.

Given these challenges, there is an urgent need for regional guidelines and consensus for the management of acromegaly within the Gulf region. Such guidelines would help standardize treatment practices, ensure consistency in clinical decision-making, and allow healthcare providers to deliver the most effective care within available resource constraints.

Consensus-driven regional guidelines, based on local resources and healthcare needs, would also improve early diagnosis and treatment, thereby preventing the long-term complications of acromegaly.

Moreover, such guidelines could help minimize the variations in practice across the region, ensuring that all patients, regardless of country, receive equitable and evidence-based care.

A working group of 15 experts from 6 Gulf countries was convened and the Delphi survey technique (10) was employed to develop appropriate guidelines and reach a consensus on the management of acromegaly in the Gulf region, considering the available healthcare resources and the challenges faced by these different countries. The goal is to provide evidence-based recommendations tailored to the specific healthcare settings of the region, ensuring standardized care while addressing variations in treatment access, availability of resources, and regional expertise.

## Methodology

### The Delphi method

To achieve this objective, the Delphi methodology was utilized to gather expert opinions and reach consensus on key aspects of acromegaly management. The Delphi method is a structured, iterative process involving a panel of experts who anonymously provide their opinions and recommendations on specific questions. This process allows for the development of evidence-based guidelines while considering the nuances of local healthcare conditions and resources. This study followed the DELPHISTAR reporting guidelines for Delphi studies, and the completed checklist is included as **Supplementary Material**.

### Participant selection

A scientific committee of five expert endocrinologists (MA, TE, KA, MM, AE) developed the study objectives and designed an online survey focusing on the treatment and follow-up of acromegaly. A panel of 15 experts in acromegaly management was selected based on their qualifications and clinical experience, representing all six Gulf Cooperation Council (GCC) countries (Saudi Arabia, UAE, Qatar, Kuwait, Bahrain, and Oman). This geographically diverse panel was assembled to ensure comprehensive coverage of regional endocrine practices. While the panel size (n=15) may appear limited, expertise and regional representation were prioritized over quantity, in alignment with Delphi methodology standards for rare diseases. Furthermore, endocrinologist with focused practice in acromegaly are limited in the region. These 15 participants are known for their expertise in acromegaly care and represent the majority of the available experts in the Gulf region. All panelists were senior endocrinologists with at least ≥5 years of acromegaly management experience, ensuring depth of insight and clinical relevance.

### Questionnaires

The initial 46 statements were developed through a structured, three-step process: First, a comprehensive literature review of international guidelines (e.g., Endocrine Society, European Society of Endocrinology [ESE]) identified core themes in acromegaly management. Second, the scientific committee adapted these themes to address Gulf-specific challenges, including disparities in healthcare resources and limited access to advanced therapies. Finally, iterative refinements were made based on panelist feedback: three statements were removed during Round 1 due to redundancy or limited regional relevance, and one additional statement was eliminated in Round 2 for the same reasons. The final questionnaire spanned six domains: primary treatment, pre-surgical SSA use, second-line therapies, radiotherapy, post-surgical follow-up, and long-term monitoring.

**Figure 1:**
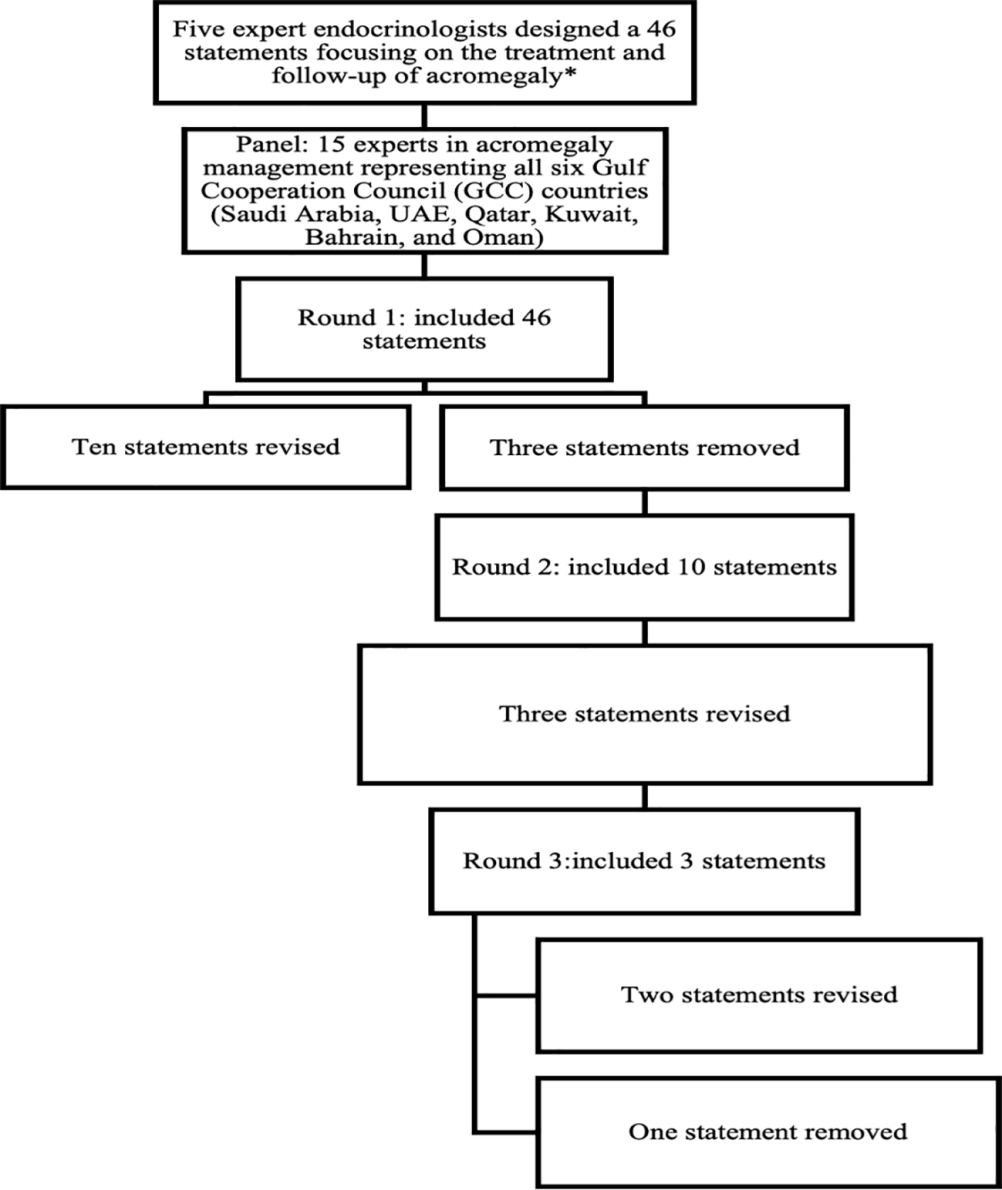
Evaluated and accepted items in each round and in the final evaluation.

Each statement in the questionnaire was rated on a 5-point Likert scale, ranging from strong agreement to strong disagreement. This enabled experts to share their insights and highlight key challenges in managing acromegaly, considering current practices, local healthcare resource constraints, and available treatment options in their country. The questionnaire was accessible through an online platform (Google Forms), and responses were collected anonymously. Consensus was defined as 66.8% or more panelists rating their agreement as either “agree” or “disagree” on the Likert scale (9). After the first round of responses, the scientific committee reviewed the feedback. Statements that did not reach the consensus threshold were revised for clarity, leading to the rephrasing of 10 statements and the removal of 3. These revisions were then resubmitted for further review in a second round on the same platform. The second round of the online Delphi survey included 10 statements from the first round. Panel members were encouraged to reconsider their initial responses considering group feedback to achieve consensus. After this round, the scientific committee reviewed the feedback. Three statements that still did not reach the consensus threshold were revised, necessitating a third and final round conducted as a virtual meeting. This meeting focused on the three statements that failed to achieve consensus in the second round, resulting in the rephrasing of two statements and the removal of one, thereby reaching the consensus threshold. The complete questionnaire, along with the consolidated responses, was sent to the entire group to represent the majority opinion, allowing experts to provide their final approval or suggestions for further refinement of the consensus. **Figure 1**. Summarize the final questionnaire domains and item attrition across Delphi rounds.

### Data analysis

Predefined criteria were established to determine the conditions under which the conclusions of the Delphi study would be considered reached. Consensus was defined as having been reached when a minimum agreement threshold of 66.8% was met (11).

## Results

The proportion of Delphi panelists that indicated some or complete agreement/disagreement with each statement is shown in **Table 1**. There was a strong consensus on referring patients with acromegaly to specialized centers with both medical and surgical expertise (93.8% agreement). Surgical intervention was overwhelmingly supported, with 100% agreement that surgery should be the preferred first-choice treatment for all eligible patients with acceptable surgical risk. Furthermore, consensus was reached that surgery remains the first-choice treatment for growth hormone-secreting pituitary adenomas both with (100% agreement) and without visual pathway compression (87.5% agreement). Moreover, 68.8% of the panelists disagreed with considering somatostatin analogues (SSA) as the first-choice treatment instead of surgery for invasive macroadenomas lacking visual pathway compression. Pre-surgery treatment with SSAs was supported by 75.0% of the panelists to be used when the surgery is delayed, and 93.8% agreed that SSAs reduce surgical and anesthesia-related risks in patients with high surgical risk such as patients with severe pharyngeal thickening, sleep apnea, or high-output heart failure. Nonetheless, there was 75% agreement against routine pre-surgical SSAs treatment due to insufficient evidence for improved outcomes. In the realm of second-line treatment, 93.8% supported watchful waiting for asymptomatic patients with marginally elevated insulin-like growth factor-1 (IGF-1) levels, and 100% agreed on initiating first-generation SSAs treatment for symptomatic patients with elevated IGF-1 post-surgery. Repeat surgery was unanimously endorsed for symptomatic patients with potentially resectable tumor remnants, while 81.3% acknowledged biochemical resistance to first-generation SSAs under specific conditions, and 93.7% agreed on recognizing radiological resistance according to defined parameters. Notably, there was substantial support (100%) for utilizing combination therapy where resources are available, notably SSAs with pegvisomant or cabergoline for patients failing to achieve IGF-1 normalization. Regarding radiotherapy, there was a 100% consensus regarding its role as a second-line treatment post-surgery for symptomatic patients with unresectable tumors and as a fallback for those unable to afford medical therapy. On the contrary, there was a 75.1% disagreement on using radiotherapy as a first-line treatment for symptomatic patients with invasive adenomas. In terms of post-surgical follow-up, experts unanimously recommended initiating the first clinical and biochemical evaluation within 1–3 months after surgery, alongside a 100% agreement on defining disease remission when post-surgery random GH levels are undetectable and accompanied by normal IGF-1 levels. There was also 93.7% consensus on adjusting SSAs dosages after the third injection based on the patient’s biochemical response. Regarding long-term follow-up, 100% of panelists supported lifelong monitoring for patients on medical treatment, while significant disagreement (68.8%) arose concerning follow-up for patients controlled by surgery alone who do not require treatment for pituitary insufficiency. Overall, these findings reflect a robust inclination toward surgical intervention as the primary treatment for acromegaly, emphasizing the importance of specialized care and long-term patient follow-up.

**Table 1:** The proportion of Delphi panelists that indicated some or complete agreement/disagreement with each statement.

Statements	Agreement
Primary treatment	
I consider referring patients with acromegaly to a specialized center with medical and surgical expertise.	93.8% agreement
I consider surgery as the preferred first-choice treatment for all eligible patients with acceptable surgical risk.	100% agreement
I consider surgery as the first-choice treatment for any growth hormone secreting pituitary adenoma without visual pathway structures compression, including both curative and debulking procedures.	87.5% agreement
I consider surgery as the first-choice treatment for any growth hormone secreting pituitary adenoma with visual pathway structures compression, including both curative and debulking procedures	100% agreement
I consider SSAs as the first-choice treatment instead of surgery, for growth hormone secreting pituitary adenoma without visual pathway structure compression, but with a low probability of complete resection (invasive macroadenomas (Knosp grade III–IV).	68.8% disagreement
Pre-treatment with SSAs	
I consider pre-surgical treatment with SSAs when there is a delay in surgery.	75.1% agreement
I consider pre-surgical treatment with SSAs to increase the likelihood of post-surgical long term disease control.	75.1% disagreement
I consider pre-surgical treatment with SSAs to reduce surgical and anesthesia-related risks (patients with severe pharyngeal thickening, sleep apnea, or high-output heart failure).	93.8% agreement
I recommend against routine pre-surgical SSA treatment due to a lack of compelling evidence for improved treatment outcomes.	75% agreement
Second line treatment	
I consider watchful waiting **for 3–6 month*s*** over initiating medical treatment in asymptomatic patients with marginally elevated IGF-I (<1.5–2 ULN) post-surgery and without significant tumor remnants.	93.8% agreement
I consider first-generation SSAs treatment post-surgery in any symptomatic patient with above normal IGF-1(>=1.5-2ULN) and an unresectable tumor residual.	100% agreement
I consider repeat surgery for in any symptomatic patient with above normal IGF-1(≥ 1.5–2 ULN) when there is a potentially resectable tumor remnant after the first pituitary surgery.	100% agreement
I consider first-generation SSAs treatment post-surgery for any symptomatic patient with above normal IGF-1(≥1.5–2 ULN) when there is no visible residual tumor on MRI.	100% agreement
I consider cabergoline treatment as first line post-surgery for any symptomatic patient with above normal IGF-1(≥1.5–2 ULN) when there is no visible residual tumor on MRI.	68.8% disagreement
I consider biochemical resistance to first generation SSAs, when the patient is on the maximum tolerated dose (usually 40 mg octreotide or 120 mg lanreotide monthly) for at least 6 months with failure to normalize IGF-I levels.	81.3% agreement
I consider radiological resistance to first-generation SSAs when the patient is on the maximum tolerated dose (usually 40 mg octreotide or 120 mg lanerotide monthly) for at least 6 months and the tumor size increases.	93.7% agreement
I consider radiological resistance to first-generation SSAs when the patient is on the maximum tolerated dose (usually 40 mg octreotide or 120 mg lanerotide monthly) for at least 12 months and the tumor size does not decrease by at least 20%.	75% agreement
I consider combination therapy where resources are available with first-generation SSAs with pegvisomant in patients with persistently above normal IGF-1(≥1.5–2 LN) while on the maximum tolerated dose of SSA (usually 40 mg octreotide or 120 mg lanerotide monthly) for at least 6 months, regardless of residual tumor size.	75% agreement
I consider combination therapy where resources are available of first-generation SSAs with pegvisomant in patients with small residual tumor after surgery, with persistently above normal IGF-1(≥1.5–2 ULN) while on the maximum tolerated dose of SSA (usually 40 mg octreotide or 120 mg lanerotide monthly) for at least 6 months.	75% agreement
I consider combination therapy of first-generation SSAs with cabergoline in symptomatic patients with IGF-1(<1.5-2ULN) while on the maximum tolerated dose of SSA (usually 40 mg octreotide or 120 mg lanerotide monthly) for at least 6 months, regardless of residual tumor size.	100% agreement
I consider combination therapy where resources are limited with first-generation SSAs with cabergoline in patients with small residual tumor after surgery, with persistently above normal IGF-1(≥1.5–2 ULN) while on the maximum tolerated dose of SSA (usually 40 mg octreotide or 120 mg lanerotide monthly) for at least 6 months.	84% agreement
I consider using second-generation SSAs for any symptomatic patient with above normal IGF-1(≥1.5-2ULN) with unresectable residual tumor that does not respond to the maximum tolerated dose of SSA (usually 40 mg octreotide or 120 mg lanerotide monthly) for at least 6 months.	87.8% agreement
I consider second-generation SSAs as the first-line treatment post-surgery for any symptomatic patient with above normal IGF-1(≥1.5–2 ULN) and unresectable residual tumors, particularly in cases of hyperintense lesions on T_2_ on MRI or adenomas with sparsely granulated pathology.	68.8% agreement
Radiotherapy	
I consider radiotherapy as first-line treatment for any symptomatic patient with above normal IGF-1(≥1.5–2 ULN) with invasive and unresectable adenoma.	75.1% disagreement
I consider radiotherapy as a second-line treatment post-surgery for any symptomatic patient with above normal IGF-1(≥1.5–2 ULN) with unresectable residual tumor.	100% agreement
I consider radiotherapy as a second-line treatment post-surgery for any symptomatic patient with above normal IGF-1(≥1.5–2 ULN) with unresectable residual tumors in cases of limited resources, when medical therapy is not available or affordable.	100% agreement
I consider radiotherapy as a third-line treatment for any symptomatic patient with persistently above normal IGF-1(≥1.5–2 ULN) on the maximum tolerated dose of first generation SSA (usually 40 mg octreotide or 120 mg lanerotide monthly) for at least 6 months and with unresectable residual tumor.	93.7% agreement
Post-surgery follow-up	
I recommend first clinical and biochemical evaluation between 1 and 3 months after surgery.	100% agreement
I recommend evaluating IGF-1 levels alone three months after surgery.	68.8% agreement
I recommend measuring random GH levels within the first week post-operatively to assess for remission.	68.8% agreement
I recommend measuring GH levels after oral glucose tolerance test (OGTT) during the post-surgical follow up only in patients with discordant IGF-1 and random GH levels at three months.	87.6% agreement
I recommend the first post-surgical imaging between 3–6 months after surgery.	100% agreement
I consider the disease in remission when the post-surgery random GH value is undetectable (<1 μg/L) with normal IGF-1 level.	100% agreement
If 3 months after surgery with apparently complete resection, the IGF-I value is >1.5 and <2 ULN, I repeat IGF-I at 1–3 months without initiating treatment.	81.3% agreement
If 3 months after surgery with apparently complete resection, the IGF-I value is >1.5 and <2 ULN, I initiate medical treatment.	74.9% disagreement
After initiating treatment with SSA, the dose is adjusted after the third injection (before the fourth dose).	93.7% agreement
In a patient with biochemical control on SSA treatment, IGF-I level determines the possible reduction in SSA dose or frequency.	100% agreement
In patients treated with surgery and radiotherapy, I consider the possibility of decreasing or suspending SSAs treatment after one to two years in order to evaluate the effects of radiotherapy.	75% agreement
Long-term follow-up after disease control	
I recommend lifelong follow-up for patients controlled on medical treatment.	100% agreement
I recommend follow-up for 5 years in patients biochemically controlled by surgery alone who do not require treatment for pituitary insufficiency.	68.8% disagreement
I recommend follow-up for 10 years in patients biochemically controlled by surgery alone who do not require treatment for pituitary insufficiency.	75.1% agreement
I recommend lifelong follow-up for patients in remission after surgery alone who do not require treatment for pituitary insufficiency.	68.8% agreement

## Discussion

Acromegaly is a rare but serious disorder, often associated with diagnostic delays and suboptimal treatment outcomes, especially in regions with limited access to specialized care (12). In the Gulf region, disparities in healthcare resources and expertise necessitate the establishment of region-specific guidelines to harmonize care and optimize outcomes. Prior studies have emphasized the variability in acromegaly management globally, with differences in access to advanced medical therapies such as first and second generation SSAs and pegvisomant (13, 14). This study bridges this gap by incorporating expert opinions from across the GCC countries, ensuring that recommendations are feasible and relevant to the region.

The findings from the Delphi consensus process provide critical insights into the management of acromegaly in the gulf region, emphasizing the need for a multifaceted and evidence-based approach. All experts (100%) agreed that surgery should be the primary treatment for eligible patients. This aligns with previous research suggesting that surgical resection of growth hormone-secreting pituitary adenomas is often the most effective means of achieving long-term disease control and improving patient outcomes (15, 16). Recent studies reinforce the notion that surgery should remain the first-line intervention for symptomatic acromegaly, particularly when dealing with tumors amenable to resection.

The expert consensus also highlighted the complexities surrounding the use of SSAs in different clinical scenarios. While 93.8% of panelists supported utilizing SSAs pre-surgery to mitigate surgical and anesthesia-related risks in high-risk individuals, there was a notable disagreement (68.8%) regarding the use of SSAs as a first-line treatment compared to surgical intervention for invasive macroadenomas. This reflects a similar trend reported in the literature, where the preference for surgical management is evident when complete resection is anticipated (3). Melmed (2018) emphasizes that while SSAs are valuable as second-line therapies or adjuncts, they are generally not recommended as substitutes for surgery, particularly in cases where surgical intervention is feasible (15).

The consensus also underscores the importance of pre-surgical and post-surgical management strategies. The agreement from 75.1% of panelists on the importance of SSAs as an interim treatment when surgery is delayed complements findings from recent studies that advocate for optimized care continuity even when immediate surgical intervention is not possible (17, 18).

However, the consensus against routine pre-surgical SSA use due to insufficient evidence for improved outcomes mirrors caution expressed in prior evaluations (19–21).

When discussing second-line treatment protocols, the expert panel reached a 93.8% consensus in favor of watchful waiting for asymptomatic patients with marginally elevated IGF-1 levels (1.5–2× the upper limit of normal [ULN]) during the early post-operative period (3–6 months). This cautious approach aligns with current guidelines advocating against overtreatment in borderline cases (22). The 1.5–2× ULN threshold was specifically adopted from the ACROPRAXIS program Delphi survey—a study on acromegaly management in clinical practice settings in Spain—which underscores the necessity of individualized decision-making in clinically ambiguous scenarios (23).

The panel agreed that radiological resistance is defined as an increase in tumor size or < 20% tumor shrinkage on MRI from baseline after receiving the maximum tolerated dose of SSA therapy for 6–12 months. This definition is consistent with what was proposed by Colao et al. (18). Patients meeting this criterion are usually less likely to achieve biochemical control with first-generation SSA alone. Therefore, consideration of add-on therapy to SSA (e.g., pegvisomant) or changing from first-generation SSA to pasireotide is an option.

The acknowledgment of biochemical and radiological resistance to SSAs reflects a refined understanding of heterogeneous treatment responses. This approach aligns with the Pituitary Society’s updated recommendations on acromegaly management, which stress the importance of vigilant monitoring and timely therapeutic adjustments in resistant cases (3). Medical therapy for acromegaly can be personalized based on patient characteristics and biomarkers to improve treatment outcomes. For example, patients with T_2_ MRI hyperintensity and specific receptor expressions (low SST2, high SST5) show better responses to pasireotide, while combination therapy with low-dose SSAs and weekly pegvisomant offers both effectiveness and cost benefits (24). Some experts consider that pasireotide can be used as first-line therapy in patients with specific characteristics, including T_2_ MRI hyperintensity, receptor expression patterns, and certain immunohistochemical patterns (25). Lim and Fleseriu (2022) further emphasized that factors such as sparsely granulated adenomas, low SSTR2 status, and genetic mutations can predict resistance to first-generation SSAs, suggesting these patients might be better candidates for pegvisomant or pasireotide treatment (26).

In the context of radiotherapy, the unanimous support for its role as a second-line treatment coincides with established literature advocating for radiotherapy in patients who do not respond adequately to surgical or medical treatments (27). This suggests that while surgery remains critical, radiotherapy plays a supportive role when other options are untenable. The panel’s disagreement regarding radiotherapy as a first-line treatment for invasive adenomas reinforces the prevailing view that surgical options should be prioritized when feasible even if the surgery will be for debulking rather than cure (28).

The expert consensus further emphasizes the necessity of structured post-operative follow-up, with unanimous support for initial biochemical evaluations within 1–3 months post-surgery. This is consistent with recent recommendations suggesting that early assessment of treatment effectiveness is crucial for guiding subsequent management and ensuring patient safety (3, 11). Additionally, defining remission based on undetectable GH levels alongside normal IGF-1 levels reflects a consensus that mirrors findings by Melmed S et al. (15), who established similar criteria for treatment success.

Finally, consensus on the need for lifelong follow-up for patients on medical treatment—while seeing significant disagreement on long-term monitoring for those managed and cured solely through surgery—highlights an area requiring further research to optimize follow-up strategies.

This Delphi consensus provides a comprehensive overview of expert opinions on acromegaly management, advocating for surgical intervention as the cornerstone of treatment while acknowledging the valuable role of adjunctive therapies. These results contribute to an evolving understanding of acromegaly management and underscore the need for tailored, patient-centered approaches in clinical practice.

While this study represents an important step toward standardizing acromegaly management in the Gulf region, it has limitations. The reliance on expert opinion may introduce bias, and the findings may not fully capture the diversity of practices across all GCC countries. Additionally, the lack of robust local data on acromegaly epidemiology and treatment outcomes highlights the need for future research to validate these recommendations in regional practice.

## Conclusion

These Delphi consensus findings provide a comprehensive synthesis of expert opinions on the management of acromegaly, highlighting the key role of surgical intervention while recognizing the importance of medical adjuncts. As the field continues to evolve, these insights reinforce the necessity for ongoing research and collaboration among clinicians to refine treatment approaches, improve patient outcomes, and tailor management strategies to individual patient circumstances.

## Data Availability

The original contributions presented in the study are included in the article/**Supplementary Material**. Further inquiries can be directed to the corresponding author.
